# Epitaxially stabilized thin films of ε-Fe_2_O_3_ (001) grown on YSZ (100)

**DOI:** 10.1038/s41598-017-02742-9

**Published:** 2017-06-16

**Authors:** Luca Corbellini, Christian Lacroix, Catalin Harnagea, Andreas Korinek, Gianluigi A. Botton, David Ménard, Alain Pignolet

**Affiliations:** 1 Centre Énergie, Matériaux et Télécommunications, INRS, 1650 boulevard Lionel-Boulet, Varennes, Québec J3X 1S2 Canada; 2 Département de Génie Physique & Regroupement québécois sur les matériaux de pointe (RQMP), Polytechnique Montréal, Montréal (Québec), H3T 1J4 Canada; 3 Department of Materials Science and Engineering and Canadian Centre for Electron Microscopy, McMaster University, 1280 Main Street West, Hamilton, Ontario, L8S 4M1 Canada

## Abstract

Epsilon ferrite (ε-Fe_2_O_3_) is a metastable phase of iron(III) oxide, intermediate between maghemite and hematite. It has recently attracted interest because of its magnetocrystalline anisotropy, which distinguishes it from the other polymorphs, and results in a gigantic coercive field and a natural ferromagnetic resonance frequency in the THz range. Moreover, it possesses a polar crystal structure, making it a potential ferroelectric, hence a potential multiferroic. Due to the need of size confinement to stabilize the metastable phase, ε-Fe_2_O_3_ has been synthesized mainly as nanoparticles. However, to favor integration in devices, and take advantage of its unique functional properties, synthesis as epitaxial thin films is desirable. In this paper, we report the growth of ε-Fe_2_O_3_ as epitaxial thin films on (100)-oriented yttrium-stabilized zirconia substrates. Structural characterization outlined the formation of multiple in-plane twins, with two different epitaxial relations to the substrate. Transmission electron microscopy showed how such twins develop in a pillar-like structure from the interface to the surface. Magnetic characterization confirmed the high magnetocrystalline anisotropy of our film and revealed the presence of a secondary phase which was identified as the well-known magnetite. Finally, angular analysis of the magnetic properties revealed how the presence of twins impacts their azimuthal dependence.

## Introduction

Nanostructures based on iron oxides are an appealing research topic within the scientific community, given the numerous applications envisioned^1–3^. The intrinsic magnetic properties of iron oxides, *e.g*. their high spontaneous magnetization at room temperature and high Curie temperature, make them suitable not only for general technological applications such as magnetic recording media or permanent magnets^4, 5^, but also for applications in various fields of medicine such as drug delivery, medical diagnostics, since beyond their magnetic properties, they are also non-toxic, biodegradable, and biocompatible^6–13^. Moreover, they are often used as models in theoretical studies to clarify particular magnetic features typical of nanoscaled systems not observable in their bulk counterparts^14^. Finally, the various forms of iron oxides constitute the most common iron compounds in nature and are generally easy to synthesize.

**Figure 1:**
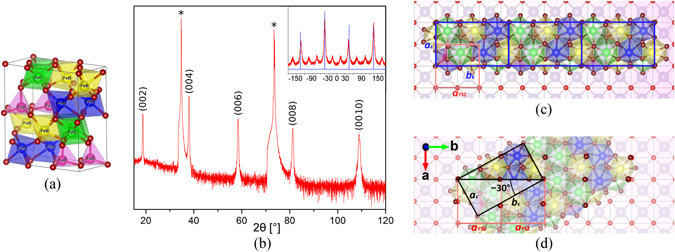
Graphic simulation of (**a**) ε-Fe_2_O_3_ unit cell, (**c**) and (**d**) the two epitaxial matches between ε-Fe_2_O_3_ (001) and YSZ (100) along with the directions of the lattice directions for YSZ. (**b**) X-ray θ/2θ diffractogram of Epsilon Ferrite thin film grown on YSZ (100), confirming the epitaxial growth. The peaks noted by asterisk are the (100) and (200) peak of the YSZ substrate. In the inset, 360° Phi scan for the (013) peak of ε-Fe_2_O_3_ (red line), along with the scan of the (220) peak of the substrate (blue), giving evidence of the formation of twinning in-plane.

Although not as easy to synthesize as the other polymorphs, due to its inherent instability in ambient condition (which however did not prevent Jian craftsmen to use it as a pigment more than 800 years ago)^15^, attention must be given to ε-Fe_2_O_3_, an intermediate phase of Fe_2_O_3_ between maghemite (γ-Fe_2_O_3_) and hematite (α-Fe_2_O_3_) with a complex orthorhombic crystal structure (Fig. 1a). Epsilon ferrite has first been synthesized in 1934, when Forestier and Guiot-Guillain reported an iron(III) oxide different from α-Fe_2_O_3_ and γ-Fe_2_O_3_^16^. Then in 1963, Schrader and Buttner^17^, and almost at the same time Walter-Levy and Quemeneur^18^, were able to synthesize the material, to measure its X-ray diffraction (XRD) pattern for the first time. Phase purity was first obtained by Trautmann and Forestier in 1965^19^. Indeed, due to its inherent structural and chemical instability in ambient conditions, ε-Fe_2_O_3_ needs to undergo size confinement, which acts as the stabilizing factor. For example, it was shown that ε-Fe_2_O_3_ could be synthesized in the form of nanoparticles encapsulated in a silica (SiO_2_) matrix by sol-gel-based methods^20^, or as nanowires^21^. These nanostructures proved to be ferromagnetic with a Curie temperature T_C_ ≈ 490 K^22^, and to exhibit remarkably high values of room-temperature coercive field, H_C_ ≈ 20 kOe^22^. The very high coercive field of ε-Fe_2_O_3_ nanoparticles makes them a promising material for non-volatile memories or permanent magnets, despite their lower spontaneous magnetization in comparison with maghemite and hematite.

X-ray magnetic circular dichroism showed that the large coercivity of ε-Fe_2_O_3_ nanoparticles originates from the presence of a large magnetocrystalline anisotropy^23^. This large magnetocrystalline anisotropy, induced by the lattice distortions of the Fe^3+^ coordination polyhedra, leads to a strong hybridization between the Fe_3d_ and the O_2p_ orbitals, resulting in a non-zero orbital magnetic moment **L**, which, through spin-orbit coupling, defines a magnetic-easy axis. Later, first principle calculations supported the experimental finding, predicting that the magnetic easy axis lies along the crystal ***a*** axis direction^24^.

Due to its high magnetocrystalline anisotropy, epsilon ferrite is one of the few materials having its natural ferromagnetic resonance (FMR) frequency in the THz range (~0.1–100 THz) frequency above 100 GHz, thus at room temperature (without applying any magnetic field). This feature is of interest considering that THz frequencies have recently received a lot of attention both in terms of fundamental physics and applications in various fields, such as communications, defense/security, medical imaging, biology and astronomy^25–29^. Moreover, since ε-Fe_2_O_3_ is characterized by an orthorhombic crystal structure belonging to the non-centrosymmetric and polar space group *Pna2*_*1*_ (Fig. 1a), it should exhibit at least pyroelectricity, which would make it a new member of the family of magneto-electric oxides^30^, or even a new room temperature multiferroic oxide. Finally, it has been recently shown how epsilon ferrite is also a very efficient catalyst for the sunlight-activated hydrogen generation from solutions of water and oxygenates^31^.

In order to integrate ε-Fe_2_O_3_ into solid-state devices, nanoparticles are usually not the most suitable form. Notably, deposition of a mixture of supported ε-Fe_2_O_3_ and ß-Fe_2_O_3_ nanorods on silicon wafer has been reported, synthesized by chemical vapor deposition^32^. However, these nanostructures do not possess the same functional properties as their nanoparticle counterparts; in particular, they exhibit a much lower coercive field. Moreover, they do not exhibit a completely ordered structure, making them unsuitable for applications where such order is needed. In order to favor integration of ε-Fe_2_O_3_ into devices and to take advantage of the magnetic, and possibly ferroelectric order(s) characterizing epsilon ferrite, the most suitable form would be *epitaxial* thin films. Recently, growth of such epitaxial epsilon ferrite thin films by pulsed laser deposition (PLD) has been reported^33^, whereby the metastable epsilon phase stabilization was accomplished by taking advantage of the epitaxial strain induced by the single crystal strontium titanate (SrTiO_3_) substrate. Growth on substrates other than SrTiO_3_, namely alumina and yttrium stabilized zirconia (YSZ), has been recently achieved, although not directly on YSZ, but with a 50 nm thick buffer layer of GaFeO_3_ (GFO) on top of YSZ used to promote the growth of epsilon ferrite^34^. Moreover, although the ferroelectric behavior of single epsilon ferrite films has never been investigated, presence of reversible spontaneous polarization was recently reported in a layered structure of SrTiO_3_:Nb/AlFeO_3_/SrRuO_3_/ε-Fe_2_O_3_^34^.

In this work, we report the growth of 100-nm-thick epitaxially stabilized epsilon ferrite thin films deposited *directly* on single crystalline 100-oriented yttrium stabilized zirconia (YSZ) using pulsed laser deposition (PLD), without the need of a GaFeO_3_ buffer layer, in contrast to previous studies^35^. YSZ(100) single crystalline substrates were chosen since they proved to promote the epitaxial growth of GaFeO_3_ (GFO)^36^, which is isostructural to ε-Fe_2_O_3_. In addition, the growth of epitaxial YSZ films on Si(100) is possible and well documented^37–39^, making it a very relevant choice of substrate for future application of ε-Fe_2_O_3_ epitaxial thin films in integrated devices. Structural characterization, comprising X-ray diffraction (XRD) and transmission electron microscopy (TEM) studies, has been carried on to confirm the epitaxial growth of the ε-Fe_2_O_3_ thin films on YSZ and to determine the epitaxy relationships between the PLD-grown films and the substrate. The characterization of the magnetic properties of the epsilon ferrite films was realized using vibrating sample magnetometry (VSM) to record their magnetic hysteresis loops for various magnetic field directions and temperatures.

## Results and Discussion

The films structure was first studied by XRD. Both classical Bragg-Brentano geometry (θ/2θ) and phi (φ) scans measurements were performed. θ–2θ scan confirmed the epitaxial growth of (001)-oriented epsilon ferrite on YSZ (100) (Fig. 1b). Given the strong K_β_ lines signal arising from the substrate, a nickel filter was used in order to cut these peaks and have a cleaner diffractogram. Prior to utilizing the Ni filter, evidences of formation of an extra phase were recorded and the extra peaks observed were attributed to magnetite (Fe_3_O_4_) (see supplementary information); given the non-epitaxial nature of the secondary phase, it is not possible, from the x-ray diffractogram, to estimate its volume relative to the main phase. It has been previously reported that in the case of GFO epitaxial films on YSZ (100) substrate, two distinct epitaxial relationships were found between GFO and YSZ^36^. Given the similitude between GaFeO_3_ and ε-Fe_2_O_3_ structures, we expected to observe a similar situation also for epsilon ferrite thin films. Such two epitaxial matches between ε-Fe_2_O_3_ and YSZ (100) are shown below in a graphical representation drawn with the software VESTA^40^, where we paid a special attention ensuring the best possible continuity between the oxygen octahedra and tetrahedra framework of the ε-Fe_2_O_3_ crystal structure and the oxygen octahedra framework of the substrate crystal structure: the first epitaxial match is given by the parallel alignment of the ***b***_**ε**_ lattice parameters of epsilon ferrite with the [100] and [010] directions of YSZ. While there is a good match between ***a***_**ε**_ and ***a***_***YSZ***_, resulting in a tensile strain of −0.58% (using *strain* = *a*_*film*_ − *a*_*sub*_ */a*_*sub*_, and the lattice parameters of ε-Fe_2_O_3_ reported for nanoparticles, *a* = 5.08 Å, *b* = 8.78 Å, and *c* = 9.47 Å as the relaxed state^41^, and *a*_*sub*_ = *a*_*YSZ*_ = 5.12 Å, as listed by the manufacturer CrysTec GmbH), the match for ***b*** is given by the so-called “3 for 5 tiling”, having 3***b***_**ε**_ ≈ 5***b***_**YSZ**_ (Fig. 1c). Note that although this epitaxy relationship gives the same sets of in-plane orientations as the one given in refs 35 and 36, we believe that the “3 for 5 tiling”, is the correct description, given the much lower compressive strain of 2.84% it introduces along the ***b*** direction compared to the description proposed in refs 35 and 36., leading to a tensile strain of −14.2%. The second match is given by the fact that the diagonal of the unit cell of ε-Fe_2_O_3_ equals approximately twice the lattice parameter ***a***_**YSZ**_ of YSZ:$\,\sqrt{{{\boldsymbol{a}}}_{{\boldsymbol{\varepsilon }}}^{2}+{{\boldsymbol{b}}}_{{\boldsymbol{\varepsilon }}}^{2}}\approx 2{{\boldsymbol{a}}}_{{\boldsymbol{YSZ}}}$. For such case, it is difficult to calculate the strain along ***a*** and ***b*** given the unusual epitaxial relation, but the tensile strain along the diagonal direction was calculated to be −0.97%. In this configuration, the angle between the directions of the ***b***_**ε**_ lattice parameter with respect to the ***a***_***YSZ***_ is equal to $\alpha ={\cos }^{-1}(2{a}_{YSZ}/\sqrt{{a}_{\varepsilon }^{2}+{b}_{\varepsilon }^{2}})\approx 30^\circ$. In both cases, the epitaxy we believe to be promoted by the continuity of the oxygen atoms framework in epsilon ferrite which align with the underlying oxygen framework of the substrate (Fig. 1d). In order to measure the three lattice parameters characterizing epsilon ferrite, and to confirm the presence of the two different twin variants of epitaxial ε-Fe_2_O_3_, the position of peaks associated with planes non-parallel to the film surface were investigated. As explained in detail in the supplementary information, the crystallographic planes chosen for determining ***a*** and ***b*** lattice parameters (***c*** was found easily from the main θ–2θ scan), were the (132) and (013) planes, respectively. While little difference was found for the position of the 013 peak, a pronounced difference was found for the 132 peak. Such evidence confirmed the presence of two distinct epitaxial variants or twins as described above (see the supplementing information for further details). Moreover, in order to prove the mutual orientation of the film and the substrate, Phi scans of the peak corresponding to the (013) plane of ε-Fe_2_O_3_ were performed along with the one of the (220) plane of the substrate, as shown in the inset of Fig. 1b (and also shown in detail in the supplementing information). Such analysis firstly confirmed the formation of twins. Furthermore, it highlighted how the peaks corresponding to the parallel direction, with a 90° symmetry, are more intense than the non-parallel ones, which appear at circa ± 30° with respect to the parallel peaks. Very similar behavior was seen also for epitaxial thin films of GFO (001) on YSZ (100)^42^.

**Figure 2:**
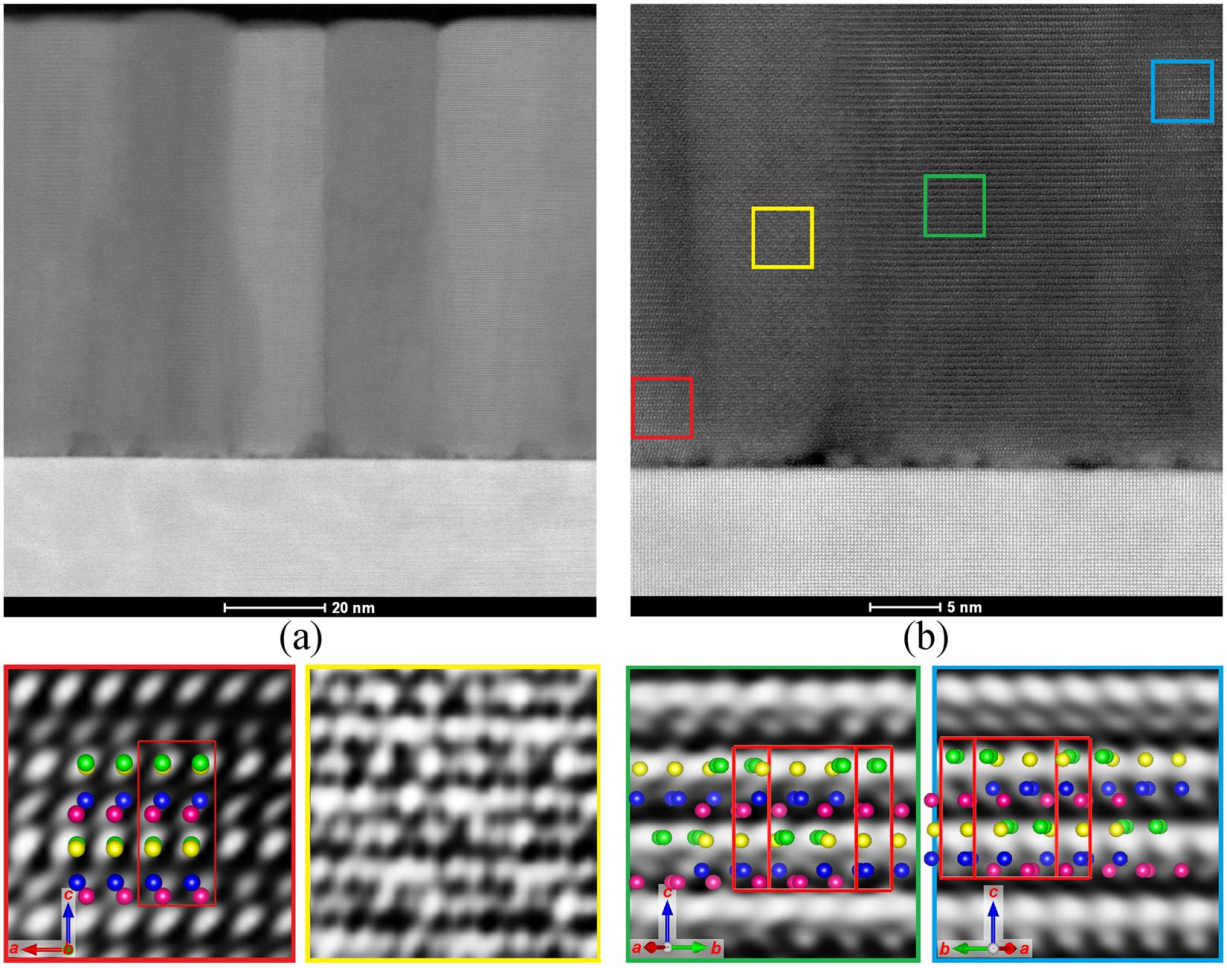
(**a**) STEM image of a circa 100 nm thick film of ε-Fe_2_O_3_ on YSZ (100) highlighting the formation of pillar-like twins, (**b**) detail of the interface between the substrate and the film, evidencing the formation of ‘bubbles’ of a foreign phase (most likely Fe_3_O_4_) at the interface. Below: high-magnification of the 4 colored selected areas overlapped with simulated ε-Fe_2_O_3_ structures rotated according to the proposed epitaxy relationships to further prove the existence of different growth in-plane orientation. If the orientation in the red box, with the b axis pointing out-of-plane is taken as 0°, the unit cells depicted in the other boxes are rotated, respectively by 300° (green), and 120° (light blue) (note that the 2 last unit cells (green) and (light blue) have a 180° rotational symmetry). No match was found for the yellow selection, probably due to the overlap of two different growth domains in the STEM image.

Aberration corrected Scanning Transmission Electron Microscopy (STEM) high-angle annular dark-field (HAADF) imaging further confirmed the epitaxial growth of epsilon ferrite on YSZ (100) (Fig. 2). In the image, we observed the presence of a thin buffer layer (few nanometers thick) at the interface between the substrate and the film. Considering that XRD has indicated the presence of a parasitic phase, which we ascribed to magnetite, we believe that this interfacial layer consists of magnetite. This will be further supported by the magnetic measurements, below. Moreover, the presence of multiple in-plane growth variants (twins) is observed (the areas with different contrast), which appear to form pillar-like structures. Finally, by selecting areas of a highly detailed picture belonging to different growth orientations and using Fourier transform enhancement software to improve the contrast of the image, we were able to pair the different structures observed in the twins with graphical unit cells rotated according to the epitaxial relationships with the substrate described above, thus convincingly confirming the proposed epitaxy relationship derived from XRD analysis. There appears to be reasonable agreement between the experimental observation and calculations of HAADF images (assuming a Gaussian probe as a first approximation) in the [010] projection of the structure in the red frame and [410] or [4$\bar{1}\,$0] for the green and blue frames. However, the agreement between the experimental pattern in the images and calculations (supplementary information) and projected structure shown, assuming the Fe ions are only visible in HAADF image, does not appear to be suitable for the area highlighted in the yellow frame, due to the possible overlap of two crystals in the projection of the foil. Nevertheless, such analysis helps to visualize the clear difference among the growth orientations and tries to match the different growth domain with the possible corresponding unit cell.

**Figure 3:**
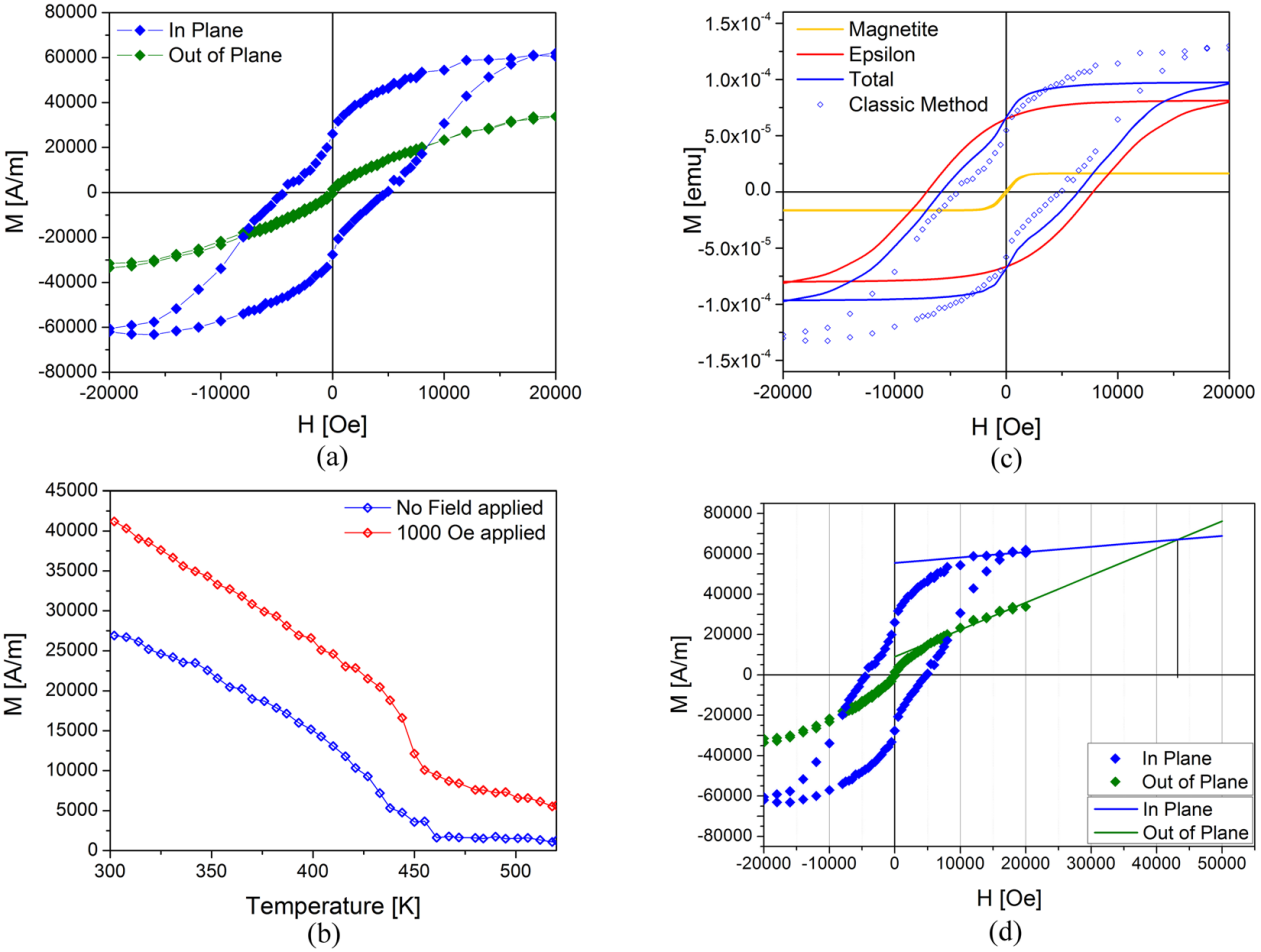
(**a**) Dependence of the magnetization of the ε-Fe_2_O_3_ thin film with the applied magnetic field in the sample plane (blue symbols) and out of the sample plane (green symbols), showing the anisotropy of the sample. (**b**) Plot of M vs T measured under no field (blue) and under an applied magnetic field of 1000 Oe (red), showing how M does not vanish for temperature higher than the Curie temperature of ε-Fe_2_O_3_ of 460 K when a small magnetic field is applied, thus revealing the presence of a soft phase with higher T_C_. (**c**) Experimental (blue symbols) and analytical (blue line) substrate corrected measurement, along with the two hysteresis loops corresponding to the separation of the epsilon ferrite (red) and magnetite (yellow) phase, respectively. (**d**) Estimation of the anisotropy field by comparing the in-plane and out-of-plane measured hysteresis loops.

The magnetic properties of the films were analyzed via Vibrating Sample Magnetometry (VSM). Hysteresis loops were first recorded with the magnetic field applied both parallel (in-plane) and perpendicular (out-of-plane) to the film surface (Fig. 3a). In order to obtain solely the film signal, magnetic contributions of the sample holder (glass rod) and the substrate were measured separately and subtracted from the total signal. The strong difference in the coercive field and in the approach to saturation at the maximum applied field between the in-plane and out-of-plane measurements confirms the high magnetic anisotropy of the epsilon ferrite epitaxial films.

Temperature dependent measurement of the remanent magnetization under no field and of the magnetization with an applied magnetic field of 1000 Oe are presented in Fig. 3b. A ferromagnetic to paramagnetic transition is observed at a temperature of ≈460 K, which we attribute to ε-Fe_2_O_3_. We note that the Curie temperature T_C_ ≈ 460 K of our ε-Fe_2_O_3_ thin films is slightly lower than the one reported for ε-Fe_2_O_3_ nanoparticles (T_C_ ≈ 490 K)^22^. Moreover, the measurements under a small applied magnetic field of 1000 Oe, well below the coercive field of the hard magnet ε-Fe_2_O_3_, but larger than the coercive field of a soft magnet, does indeed reveal the presence of a soft secondary ferromagnetic phase with a T_C_ higher than 550 K, consistent with the presence of some Fe_3_O_4_ (Tc = 850 K) observed by XRD. In contrast to some explanations reported in the literature resorting to the presence of multiple twins to explain the pinched nature of the loop, and as we will see below, the pinched nature of the hysteresis loop can be simply explained by the simultaneous presence of epsilon ferrite and magnetite. In order to determine the relative contribution of the two magnetic phases ε-Fe_2_O_3_ and Fe_3_O_4_ to the measured magnetic hysteresis loop, we developed a method consisting in differentiating the measured loop, fitting the various peaks of the 1^st^ derivative by using a summation of Voigt distributions in addition to a constant, deconvoluting the signals originating from the different magnetic phases (which simply correspond to the different peaks), and finally integrating exclusively and independently each Voigt functions^43^. Our technique, which we named D-D-SI, from “Derivative - Deconvolution - Selective Integration”, allows us discriminate the various contributions originating from different magnetic phases, and also to eliminate every linear contribution due to the paramagnetic and/or diamagnetic signals arising either from the vibrating rod or/and from the substrate, along with any further linear contribution possibly coming from the film.

The reconstructed loops obtained from the D-D-SI technique are shown in Fig. 3c for the two ε-Fe_2_O_3_ and Fe_3_O_4_ magnetic phases (red and yellow lines) and the sum of the two magnetic contributions (blue line). For comparison, the hysteresis loop obtained using the conventional way, *i.e*. by subtracting the measured contributions of the substrate and the rod from the measured signal of the thin film is also shown (blue symbols). In order to compare the two loops and demonstrate that the experimentally measured loop is well described by their sum (the small residual being a linear contribution that has not been captured in the modeling), we show the moment, as measured [emu] rather than the magnetization (moment/volume). Plotting the magnetizations in SI units [A/m] (see Figure SI 4), would make this comparison and graphical addition more difficult given the higher magnetization of magnetite compared to epsilon (3–4 times higher) and the different volumes of the soft magnetite and hard epsilon ferrite phases, which ratio was estimated to be circa 1:10. The volume ratio was found assuming the magnetite phase to be in the form of nanoclusters and its magnetization to be 309 [emu/cm^3^]^44–48^. It has to be noted that utilizing such value for magnetization, instead of the standard value for bulk magnetite of 480 [emu/cm^3^]^49^ results in a higher estimation of the volume ratio (estimated to be 1:10), which would be 1:15 if the value of bulk magnetite is used. As discussed in ref. 43, the difference observed between the hysteresis loops obtained using the D-D-SI method and the hysteresis loop obtained using the conventional method corresponds to a linear contribution. This linear contribution can originate from different sources. For example, an error measurement of 7.5% on the magnetic contribution of the substrate could explain the difference^43^. Alternately, the difference could also by explained by the presence of ferromagnetic domains in the thin films, whose hard axis is parallel to the applied field direction.

Our results indicate that the presence of the magnetite parasitic phase, which possesses a coercive field of ~100 Oe, has the drastic effect of reducing the coercive field of the hysteresis loop of the whole film (Hc ~ 5 kOe). In comparison, the epsilon ferrite domains are individually characterized by a magnetization at saturation of M_s_ ≈ 40 kA/m and a coercive field H_C_ ≈ 8 kOe (see supplementary information for the method for determination of M_s_ in S.I. units). Furthermore, by extrapolating the values in the linear regime for both the in-plane and out-of-plane hysteresis, we estimated the anisotropy field to be ~43 kOe (Fig. 3d). We note that these values are smaller than those reported in the case of epsilon ferrite nanoparticles (M_s_ ≈ 15–20 emu/g, which gives M_s_ ≈ 81–108 kA/m using 5.4 g/cc as density for epsilon, H_C_ ≈ 20 kOe and H_A_ ≈ 65 kOe). The lower coercivity measured can possibly be explained by the presence of multiple growth variants, each with its easy axis in a different direction, thus averaging the magnetic hysteresis loops along several magnetic easy axis. As a consequence, it is possible that our thin films are not completely saturated when subjected to a field of 20 kOe, which is the maximum field of our VSM. As a consequence, the magnetization at saturation, the coercive field and anisotropy field values obtained here can be considered as lower limits.

Finally, we note that a pinched hysteresis loop was also observed in epitaxial (001)-oriented thin films of epsilon ferrite grown on different (111)-oriented perovskites (SrTiO_3_, LaAlO_3_ and LSAT) by our group (unpublished), as well as in epsilon ferrite thin films synthesized by other groups on SrTiO_3_ (111)^33, 34^. We note also that other effects may contribute to the pinched nature of the magnetic hysteresis loop, such as the presence of antiphase boundaries (APB) between the differently oriented growth domains^50^, where the magnetization may, for example, lie perpendicular to the film surface, or the presence of nanostructures which may grow on top of the film, as reported for CoFe_2_O_4_ epitaxial thin films^51^. However, we believe that the simplest manner to explain it, overlooked so far in the published literature, is the presence of a soft magnetic phase in addition to epsilon ferrite.

**Figure 4:**
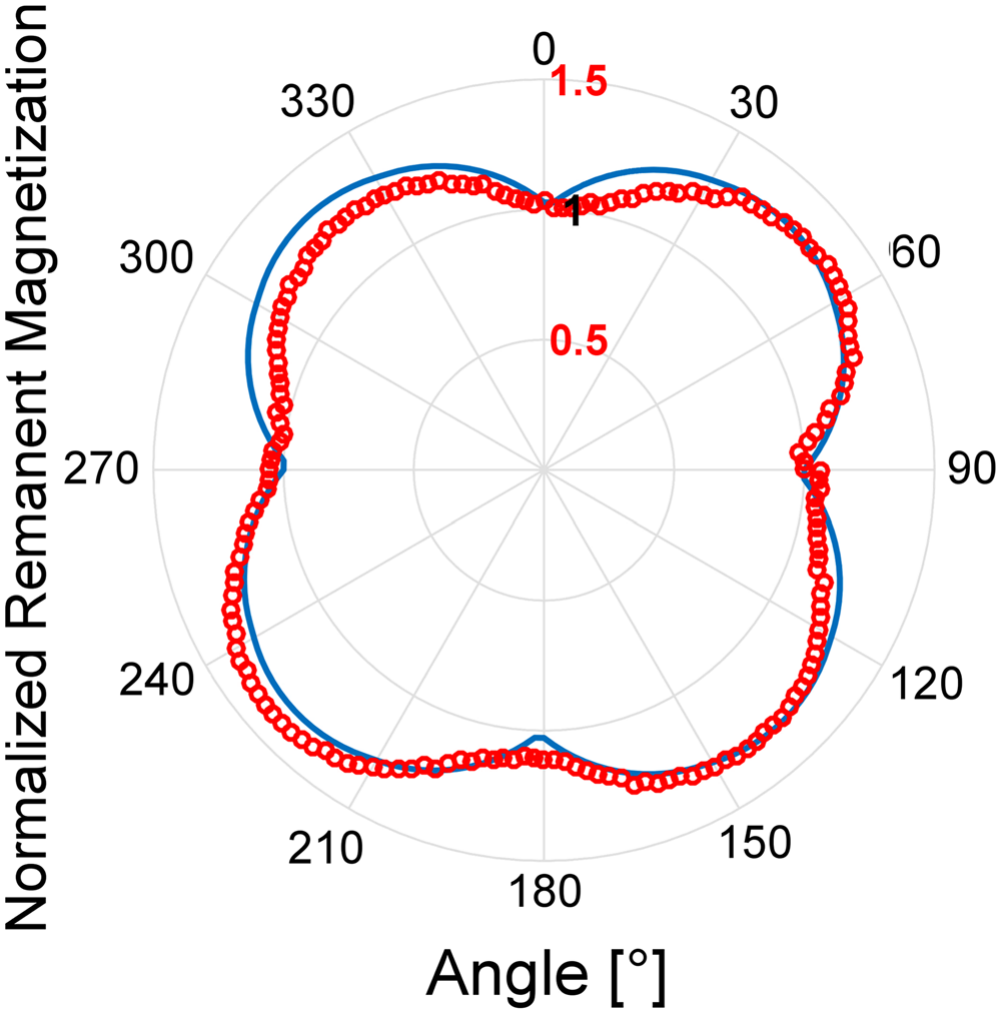
Polar plot of normalized M_r_ vs angle (red symbols), and the model we developed to fit the data (blue line).

The angle-dependent magnetic properties was obtained by recording in-plane hysteresis loops every 2 degrees. The in-plane angle dependence of the remanent magnetization M_R_ was extracted from the measured loops. To analyze the azimuthal dependence of M_R_, a simple model which averages the remanence of the different growth variant parallel to the measured axis was utilized^52^. Such model is based on the assumption that, at zero applied field, the magnetization inside each crystal is uniform and aligned along their magnetic easy axis (*a*–axis). This assumption is supported by a critical dimension analysis based on the anisotropy field values and the dimensions of our epsilon ferrite crystals obtained from TEM (see Fig. 2) allowing us to infer that the crystals are essentially ferromagnetic monodomains^30, 52^. The angular dependence of the normalized remanent magnetization for one crystal orientation can thus be modeled by the absolute value of *cos(angle* + *offset)*. Six different contributions to the remanent magnetization were used corresponding to the six different growth variants (two “parallel” with ***bε*** aligned along the [010] and the [100] direction of the substrate, and four “non-parallel”, aligned along the $[1\bar{1}0]$, $[4\bar{1}0]$, [410], and [110]). The contribution of each variant to the total was weighted in order to fit satisfactorily the measured data with our model (see Fig. 4). Our results indicate that the magnetic contribution of the two “parallel” variants account for the larger contribution (~93% of the total remanent magnetization), which is consistent with the data found by angular XRD (~85% of the counts, inset of Fig. 1b and supplementary information). The difference between the measured data and the model can be attributed to the non-concentric rotation of the rod holding the sample during the measurements^53, 54^.

Finally, using our D-D-SI method, we extracted the in-plane angular dependence of M_R_ for the magnetite phase. Our results show that the remanent magnetization of magnetite do not significantly varies with the angle at which the magnetic field is applied (not shown; see supplementary information).

## Conclusions

We successfully grew (001)-oriented epitaxial thin films of epsilon ferrite on single crystalline YSZ (100) via pulsed laser deposition. As seen for epitaxial thin films of GaFeO_3_, isostructural to ε-Fe_2_O_3_, YSZ (100) proved to promote two different epitaxy relationships. Moreover, due to the lower symmetry of the film in comparison to that of the substrate, the growth lead to multiple positioning or twinning, which was observed and confirmed both through x-ray diffractometry and TEM imaging. These features lead to an angular dependence of the magnetic properties that may represents an issue in uses where a single magnetic easy axis is required, like in magnetic memories. For application in such devices, epitaxial films of epsilon ferrite with a single in-plane orientation need to be grown. Nevertheless, the ability of growing ε-Fe_2_O_3_ epitaxially on YSZ (100) is of great significance, given how this material is often used as a buffer layer for the growth of oxides on top of (100)-oriented silicon wafers^37–39^.

Along with the desired ε-Fe_2_O_3_ phase, concomitant growth of randomly oriented magnetite was recorded both via XRD measurements and trough magnetic characterization. The presence of the soft magnetic phase ‘magnetite’ correctly explains the pinched nature of the magnetic hysteresis loops measured. Data processing, allowed us to separate the contribution of the two phases from the total magnetic hysteresis. Although non-ideal, formation of a soft magnetic phase should not necessarily constitute a problem for all magnetic applications; in particular, devices based on ε-Fe_2_O_3_ for THz applications will not be affected given the much lower FMR frequency of the soft magnetite.

## Methods

### Growth of thin films

The films were deposited by pulsed laser deposition utilizing a Kr excimer laser (wavelength 248 nm, pulse duration ~22 ns) with a pulse repetition rate of 5 Hz, focused on the surface of a 1 inch diameter ceramic target placed circa 5.5 cm far from the substrates with a fluence of ~1.8 J/cm^2^ (spot size ≈ 0.03 cm^2^). The target was prepared by sintering Fe_2_O_3_ powders (Semiconductor Industries, 99.9% purity), resulting in a mixture of polycrystalline hematite (α-Fe_2_O_3_) and amorphous Fe_2_O_3_. The thin film growth was performed at substrate temperature of 800 °C in oxidizing environment, where the O_2_ pressure was maintained at 75 mTorr. Thickness of the films was measured by profilometer and Atomic Force Microscopy (AFM), yielding a growth rate of ~0.04 Å/pulse.

### Structural characterization

Structural characterization was performed by X-ray diffraction using the K_α_ radiation of copper (λ = 1.5407 Å) in a PANalytical X’Pert MRD PRO diffractometer.

Cross-sectional transmission electron microscopy samples were prepared with a Focused Ion Beam instrument (Zeiss NVision 40) with multiple milling stages at decreasing incident ion energies down to 5 keV, and a final polishing step using an Ar-beam milling using a Fischione NanoMill operated at liquid nitrogen temperatures as discussed in detail in a previous report^55^. This procedure has been shown to minimize damage and oxygen loss even in beam-sensitive samples.

Electron microscopy observations were carried out with an FEI-Titan 80–300 Scanning Transmission Electron Microscope (STEM) equipped with aberration correctors of the probe and imaging forming lenses, an energy loss spectrometer (Gatan Quantum model), and an electron beam monochromator. Imaging with a high-angle annular dark field detector and collection angles providing atomic-number (Z) contrast, was used to provide information on local structural arrangement, including the interface atomic structure and the presence of grain boundaries separating the crystalline grains in the films.

### Magnetic characterization

An EV9 Vibrating Sample Magnetometer (VSM) made by ADE Technologies (MicroSense, Lowell MA, USA) was used to characterize the magnetic properties of the samples. Magnetic hysteresis loops were recorded for the epsilon ferrite thin films at room temperature with the magnetic field, varying between ± 20000 Oe, applied both parallel (in-plane) and perpendicular (out-of-plane) to the film surface.

## Electronic supplementary material

Supplementary Information
