# Supplementary material for: Epitaxially stabilized thin films of ε-Fe2O3 (001) grown on YSZ (100)
Source: Sci Rep. 2017 Jun 16;7:3712. doi: 10.1038/s41598-017-02742-9 (PMC5473884; doi:10.1038/s41598-017-02742-9)
Supplement: Supplementary file 1 — Supplementary Information [file 41598_2017_2742_MOESM1_ESM.pdf]

Epitaxially stabilized thin films of ε-Fe_2O_3 (001) grown on YSZ (100) – Supplementary Information

Luca Corbellini[1], Christian Lacroix[2], Catalin Harnagea[1], Andreas Korinek[3], Gianluigi A. Botton[3], David Ménard[2] and Alain Pignolet[1].

[1]Centre Énergie, Matériaux et Télécommunications, INRS, 1650 boulevard Lionel-Boulet, Varennes, Québec J3X 1S2, Canada
[2]Département de Génie Physique & Regroupement québécois sur les matériaux de pointe (RQMP), Polytechnique Montréal, Montréal (Québec) H3T 1J4, Canada
[3]Department of Materials Science and Engineering and Canadian Centre for Electron Microscopy, McMaster University, 1280 Main Street West, Hamilton, Ontario L8S 4M1, Canada

1. Presence of a second soft magnetic Fe_3O_4 phase

As reported in the main body of the manuscript, a careful scrutiny of the x-ray diffraction spectrum revealed the presence of peaks belonging neither to the main investigated phase ε-Fe_2O_3 nor to the substrates and its secondary lines ($K\alpha_2$, Kß, or the L line of the tungsten contamination of the tube, which are essentially cut in the diffractogram utilizing a Ni filter). Such peaks were identified as polycrystalline cubic magnetite (Fe_3O_4), which means the presence of iron Fe^{2+} not fully oxidized ions of which is both likely and is consistent with the magnetic data recorded.

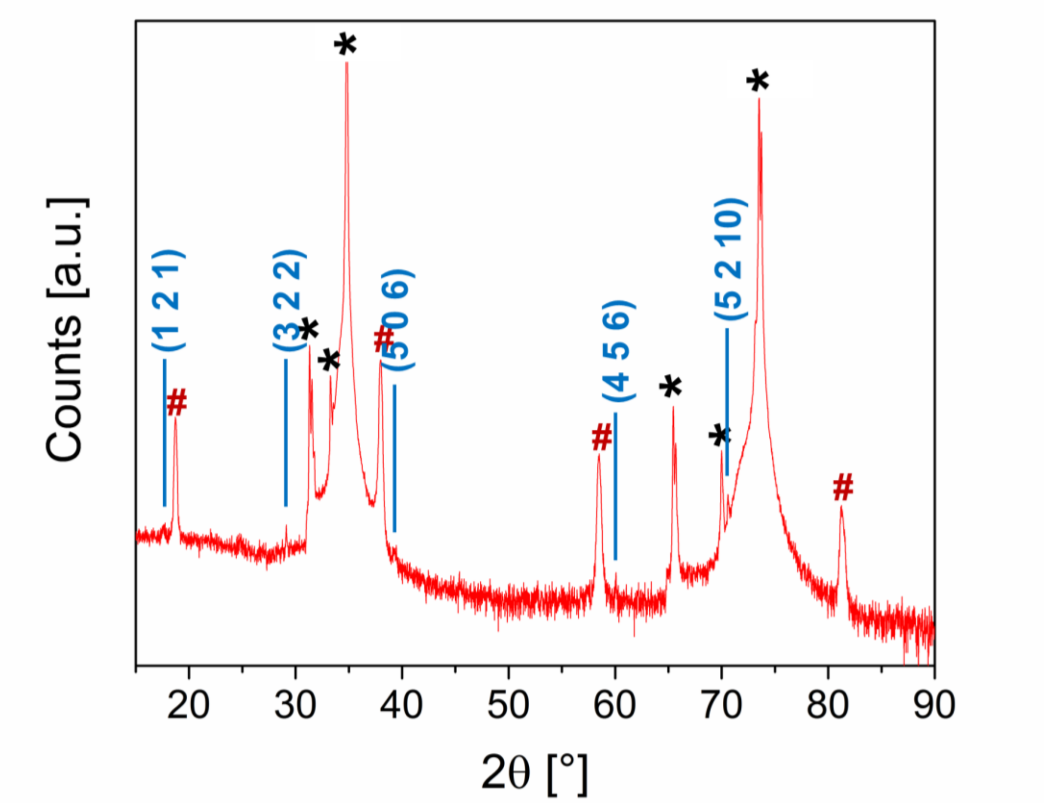

Figure S1: θ-2θ diffractogram before the application of the Ni filter, on which the peaks corresponding to the extra phase (marked by blue lines and indexed with the corresponding plane) and identified as magnetite are clearly visible. The peaks indexed with a black asterisk (*) belong to the YSZ (100) substrate and its secondary lines, while the one marked by a magenta pound (#) belong to the epsilon ferrite (001) film.

2. Determination of the lattice parameters for the two growth twins observed

Once confirmed the fact that ε-Fe_2O_3 grows epitaxially on YSZ (100) single crystal substrates, peaks associated to crystallographic planes non-parallel to sample surface were investigated in order to estimate the lattice parameter of the unit cell in the plane of the film. The planes chosen for determining ***a*** and ***b*** lattice parameters (***c*** was found easily from the main θ-2θ scan), were the 132 and 013, respectively. The initial values of the 2θ angles, to be further refined, were found by simulating the powder diffraction pattern of a epsilon ferrite unit cell with atomic coordinates and the values of the ***a*** and ***b*** lattice parameters of the unit cell of ε-Fe_2O_3 reported for nanoparticles (obtained by Rietveld refinement), together with the value of ***c*** experimentally measured. This allowed us to estimate the value for both the position (θ/2θ) of the peaks, and the angle between the two planes considered and the surface (χ). For the 013 family, the angle between the planes and the surface was set to $\chi = 19.89°$, while fore the 132 family $\chi = 62.58°$. Furthermore, the scans were taken at different values for Phi (ϕ), following the angular relation found in the phi scan of the 013 planes (Figure S3). While no major variation was found for the 013 peak, two different sets of peaks corresponding to the 132 plans were found (Figure S2). This suggests that, while the ***b*** lattice parameter is always the same, the ***a*** lattice parameters has two different values depending on the in-plane orientation.

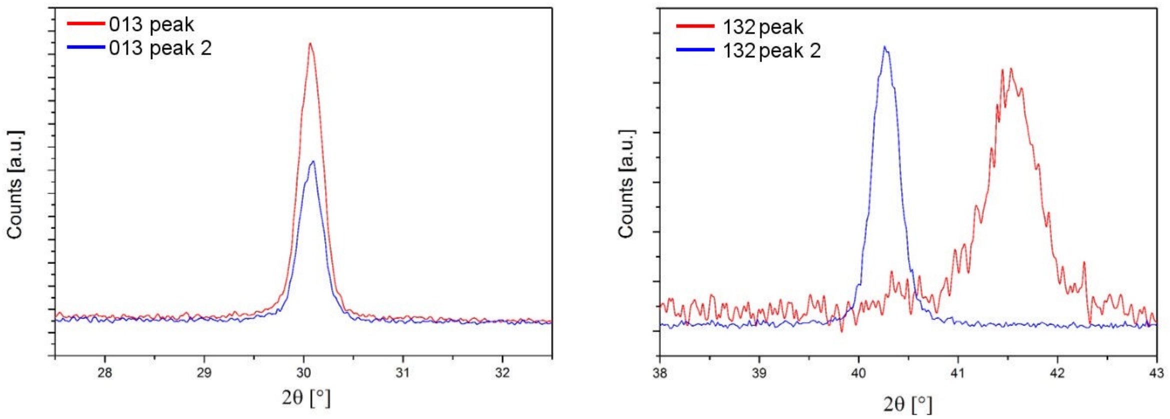

Figure S2: θ-2θ scan around the peaks associated with the (013) and the (132) planes of epsilon ferrite. The two different variants for the 132 peak confirm the double epitaxy of epsilon ferrite on YSZ (100).

From the positions of the peak recorded, we were able to estimate the lattice parameters from the 2θ value of the two peaks and compare them with the epitaxial relation we proposed, as shown in the table below. The smaller lattice parameter (noted by "1"), belongs to the "non-parallel" epitaxial match, while the larger one ("2"), corresponds to the "parallel" one, in complete agreement with the phi-scan of the 013 peak presented in the inset of Fig.1 and presented in Fig. S3.

	a_1	a_2	b_1	b_2	c
Measured Parameter [Å]	4.73	5.11	8.69	8.73	9.47

Table 1: Calculated lattice parameters for the two epsilon ferrite variants growing on YSZ (100).

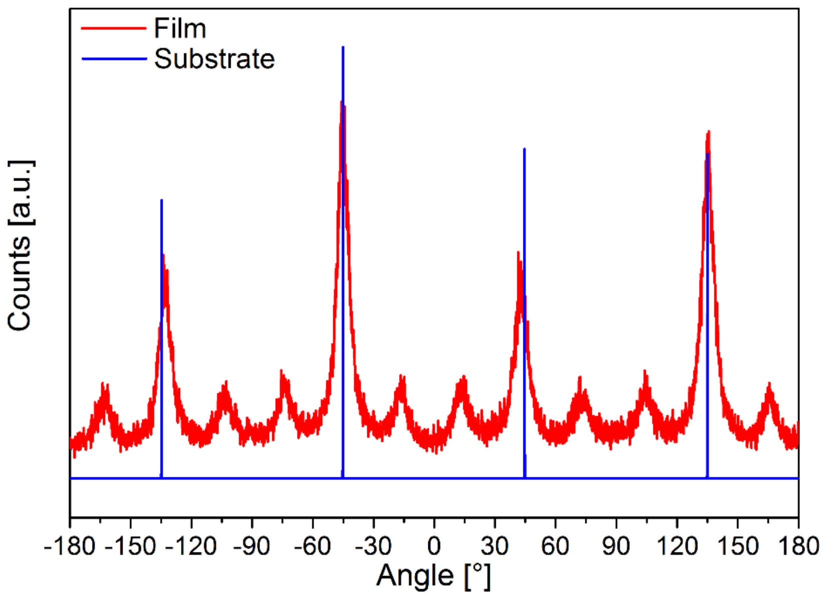

Figure S3: Plot of the phi-scan of the 013 peak of the film along with the 220 peak of the substrate.

3. Detailed analysis of the transmission electron microscopy images and simulations

In order to obtain some insight of the Scanning Transmission Electron Microscopy results, multi-slice calculations of high-angle annular dark field (HAADF) images were carried out in different projections corresponding to possible orientation expected from X-ray diffraction data. While it is possible that some residual probe aberration and crystal alignment would affect the detailed contrast, the results from the simulations showed reasonable agreement for the region highlighted in "red" (corresponding to the [010] projection) and for the “green” and “blue” selections (corresponding to the [4 $\bar{1}$ 0], [4 1 0] with some sample tilt) (see Figure 2 and Figure S4), but no satisfactory agreement with the "yellow" projection in Figure 2. For this latter case, the lack of correspondence between the experimental and simulation is likely due to the overlap of two areas with different growth variants within the thickness of the thin foil.

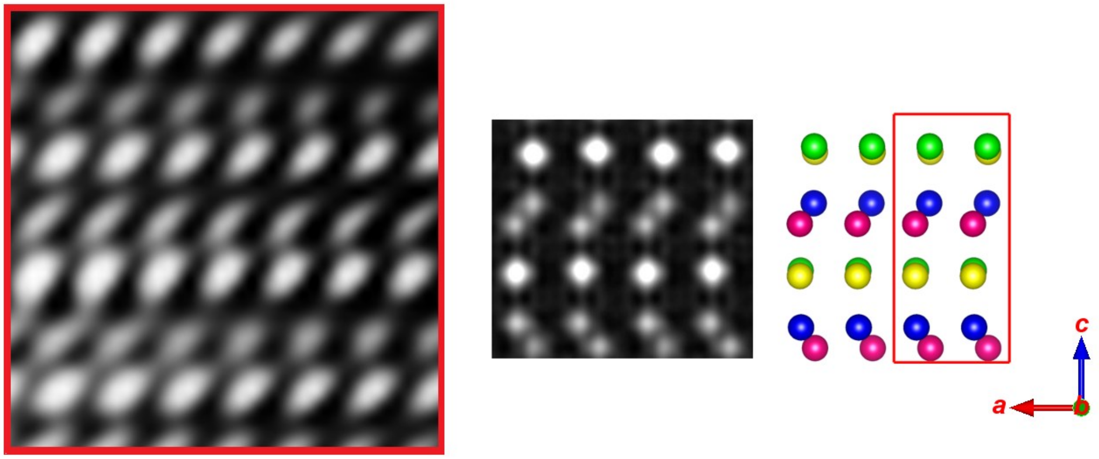

(*a*)

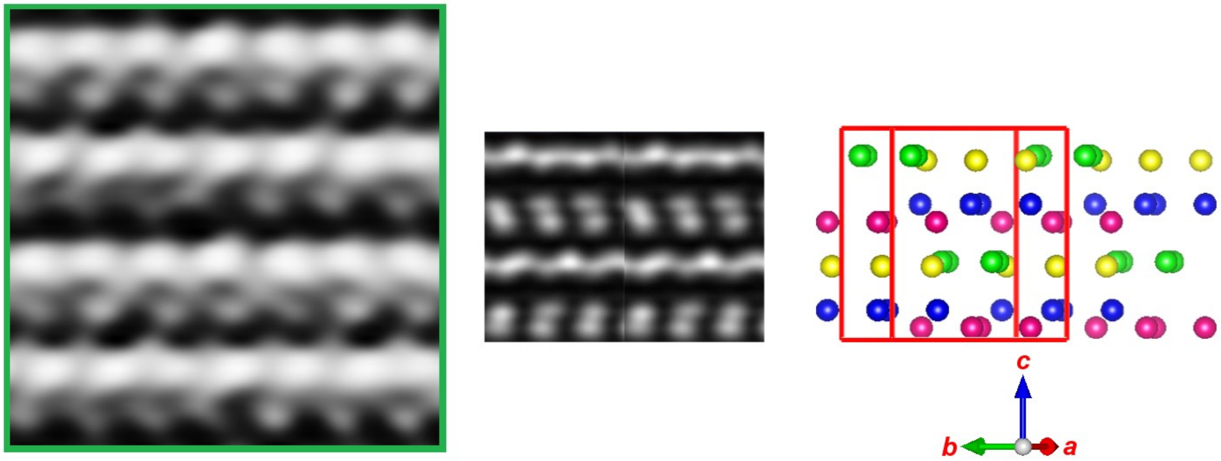

(*b*)

Figure S4: Selections of an STEM figure of a (001)-oriented thin film of epsilon ferrite grown on YSZ (100). Highlight, simulation of the STEM pattern, and graphical simulation of an (a) [010] *a (b)* [4 $\bar{1}$ 0] (or [4 1 0]) *oriented growth domains.*

4. Individual magnetization of the ε-Fe_2O_3 and Fe_3O_4 phases

In order to separate the magnetization curve for each phase, we estimated the volume of each phase (ε = epsilon ferrite ε-Fe_2O_3 and Mag = magnetite Fe_3O_4) using the following methodology:

- Estimation of the total volume of our sample by knowing the growth rate (thus the thickness *t*) and measuring the surface area (*S*) of our films: $V_{Tot} = t*S$;
- The values of the total magnetic moment in [emu] for epsilon ferrite and magnetite were found by using the "D-D-SI" method, extracting the two individual contributions (ref. 43);
- The volume of magnetite was estimated by taking the normalized magnetization value reported in literature for Fe_3O_4 nanostructures $M_s = 309$ [emu/cm^3] and using $V_{Mag} = M_s$ [emu] (of the film)/M_s [emu/cm^3] (in literature). It is important to note how the magnetization for bulk magnetite is considerably higher ($M_s = 480$ [emu/cm^3]), and using such value for the volume estimation would lead to a value smaller by 35%;
- The volume of epsilon was the found by subtracting the Magnetite volume from the total: $V_\varepsilon = V_{Tot} - V_{Mag}$;
- By using V_ε and V_{Mag} (which are circa 10:1 in ratio), the magnetization in [A/m] for the each phase was found.

With this procedure, we found that the magnetization at saturation of epsilon ferrite to be $M_s \approx$ 40 kA/m.

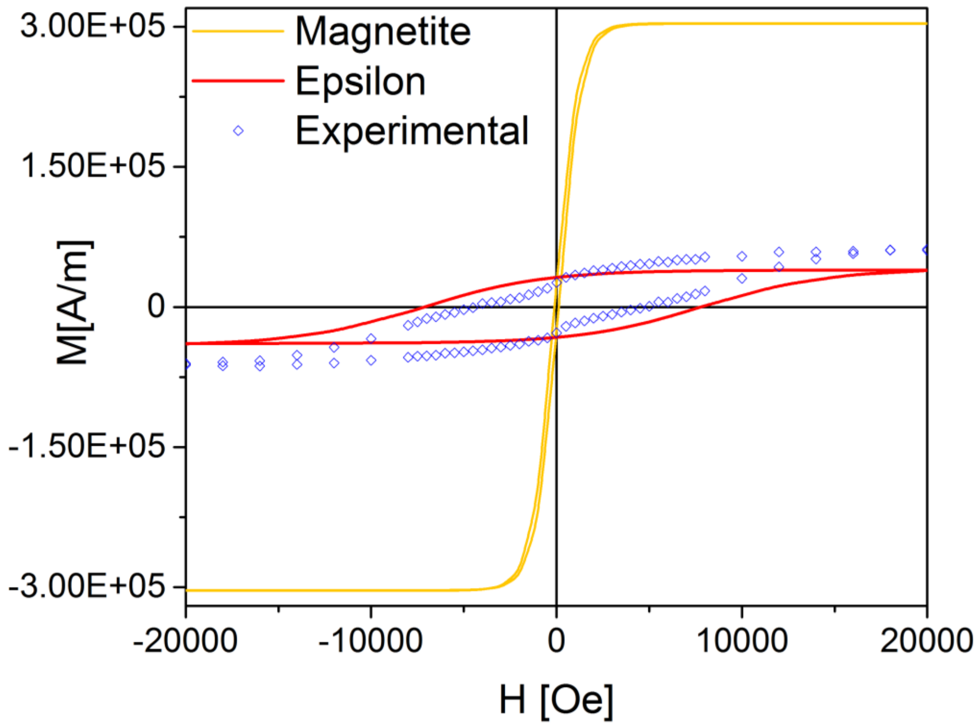

Figure S5: Plot of the magnetization curves for magnetite and epsilon ferrite expressed in A/m. It has to be noted that in normalized units ([emu/cm^3] in CGS or [A/m] in SI), the experimental data is not fitted by the sum of the loops for ε-Fe_2O_3 and Fe_3O_4 as it was the case in Fig. 3, since both the volume fraction and the M_s of the two phases are very different.

5. Angular Dependence of the magnetic properties

In order to explain the angular dependence of the magnetic properties, a simple model, based on the epitaxial matches between epsilon ferrite and the YSZ (100) substrate and the Stoner-Wohlfarth model, was developed. First, the different growth orientations for epsilon ferrite on YSZ (100) were identified. Six different growth variants (two “parallel” with ***b*** aligned along the [0 1 0] and the [1 0 0] direction of the substrate, and four “non-parallel”, aligned along the [1 $\bar{1}$ 0], [4 $\bar{1}$ 0], [4 1 0], and [1 1 0]) were found (see Fig. 1). According to the Stoner-Wohlfarth model, the angular dependence of the remanent magnetization of a single-domain can be modeled using the absolute value of $cos(x+\varphi)$, where x is the angle between the applied magnetic field and the YSZ [010] direction and φ is the angle between the b-axis and the YSZ [010] direction. We note that the normalized remanent magnetization (M_r) of a growth variant is then 1 when the magnetic field is applied along its magnetic easy axis, and 0 when the magnetic field is applied along its hard axis. Therefore, the total angular dependence of M_r can be expressed as:

$$M_{R\,Total} = x(1) * abs\,(\cos(x)) + x(2) * abs\,\left(\cos\left(x - \frac{\pi}{2}\right)\right) + x(3) * \left[abs\,\left(\cos\left(x - \frac{\pi}{6}\right)\right) + abs\,\left(\cos\left(x - \frac{\pi}{3}\right)\right) + abs\,\left(\cos\left(x - \frac{2\pi}{3}\right)\right) + abs\,\left(\cos\left(x - \frac{5\pi}{6}\right)\right)\right], \qquad \text{Equation 1}$$

where x(1), x(2) and x(3) correspond to weight factors representing the volume fraction for each growth variant: x(1) corresponds to crystals whose b-axis is oriented in the YSZ [010] direction, x(2) corresponds to crystals whose b-axis is oriented in the YSZ [100] direction and x(3) corresponds to the “non-parallel” crystals. We assumed that the weight factor is the same for all “non-parallel” crystals in agreement with XRD data (see Fig. S3).

Our results indicate that the magnetic contribution of the two “parallel” variants account for the bigger contribution (~93% of the total remanent magnetization), which is consistent with the data found by angular XRD (~85% of the counts, Figure S3).

Finally, we drew in the same polar plot M_r versus angle and the phi-scan of 013, together with the graphical representation of the six variants (twelve are shown due to a 180° symmetry) in the background.

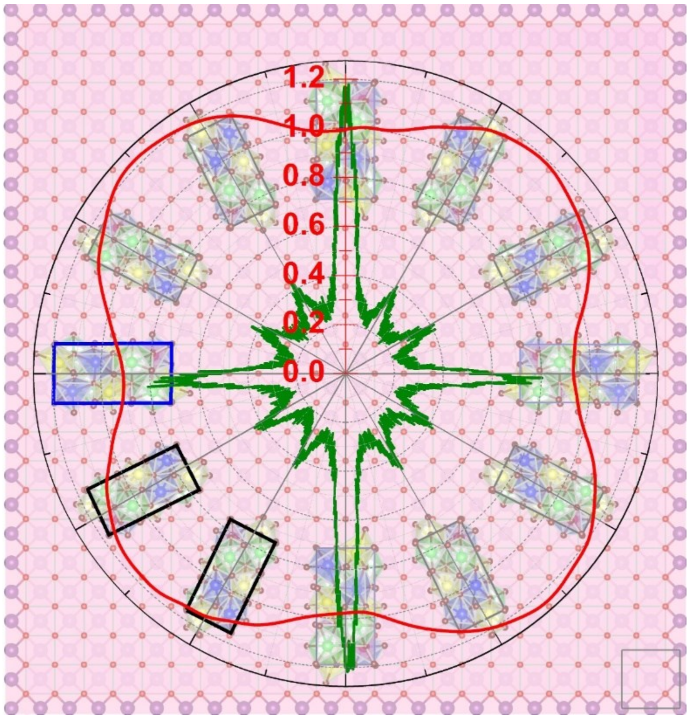

Figure S6: Polar representation of the (013) XRD phi-scan for the epsilon ferrite 013 peak (green line) along with the azimuthal dependence of M_R (red line), overlapping the graphical simulation of twinning for ε-Fe_2O_3 thin film grown on YSZ (100).

In Figure S7, we compare the angular dependence of the relative remanent magnetization of each phase, epsilon (in red) and magnetite (in yellow) extracted using the D-D-SI method. We observe that no angular dependence is detected for magnetite, which is expected considering that magnetite does not possess a large magnetocrystalline anisotropy like epsilon ferrite.

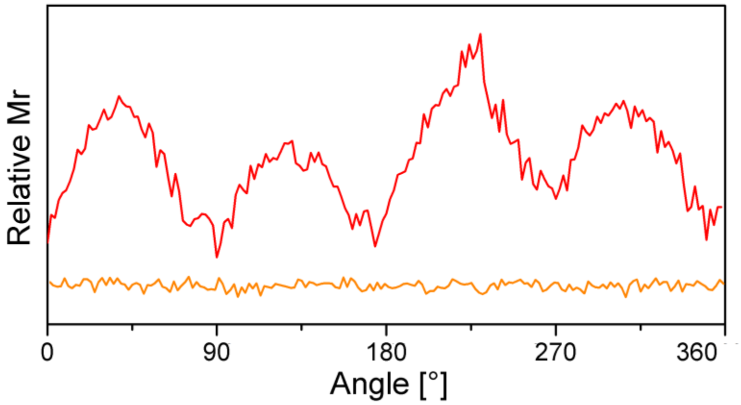

Figure S7: Plot of the relative remanent magnetization M_r in function of the angle for the epsilon phase (in red) and the extra magnetite phase (in orange). The two curves are shown with different scale to emphasize the angular dependence of the remanent magnetization of the epsilon phase in contrast to the constant and isotropic remanent magnetization of the magnetite phase.
